# Case Report: Cerebral Venous Sinus Thrombosis in a Young Child With SARS-CoV-2 Infection: The Italian Experience

**DOI:** 10.3389/fneur.2022.861345

**Published:** 2022-03-31

**Authors:** Paola Silvestri, Anna Clemente, Alberto Spalice, Alessandra Febbo, Luigi Matera, Fabiana Accardo, Maria Antonietta Barbieri, Alberto Villani, Fabio Midulla

**Affiliations:** ^1^Maternal-Infantile and Urological Science Department, Sapienza University of Rome, Rome, Italy; ^2^Department of Pediatric Emergency Medicine, Bambino Gesù Children's Hospital, IRCCS, Rome, Italy; ^3^Academic Department of Pediatrics, Bambino Gesù Children's Hospital, IRCCS, Rome, Italy

**Keywords:** cerebral thrombosis, SARS-CoV-2 infection, COVID-19, pediatric, stroke

## Abstract

**Introduction:**

Severe acute respiratory syndrome coronavirus 2 (SARS-CoV-2) infection in pediatric patients is usually characterized by fever, dry cough, and fatigue, or is asymptomatic and rarely presents with pneumonia. On the other hand, cerebral venous sinus thrombosis (CVST) could be a neurological sequela of the prothrombotic state triggered by coronavirus disease 2019 (COVID-19) both in adults and children.

**Case Report:**

We present a case of a 15-year-old male child who was obese and had mild neurocognitive impairment. He was admitted to the pediatric emergency department and then diagnosed with CVST during SARS-CoV-2 infection.

**Conclusion:**

During the COVID-19 pandemic, in patients presenting with neurological manifestations of CVST (headache, alteration of consciousness, focal deficit, or signs of endocranial hypertension), it is advisable to look for a current or recent infection of SARS-CoV-2, regardless of the presence of respiratory symptoms. In our patient, ongoing SARS-CoV-2 infection represents the only prothrombotic risk factor underlying the neurological disease.

## Introduction

The first SARS-CoV-2 outbreak was described in Wuhan, China in December 2019, and on March 2020, it was declared a pandemic by WHO (1). In pediatric age, SARS-CoV-2 infection is frequently associated with a less severe clinical picture, if compared to adults (2, 3). In fact, majority of pediatric patients with SARS-CoV-2 infection present with fever, dry cough, and fatigue, or are asymptomatic and rarely have pneumonia (3); 2–6% of children manifest severe illness (4). Thrombotic complications can worsen the clinical course of an infection (5). In patients with SARS-CoV-2, acute ischemic stroke incidence is about 2.3% (5, 6), and most cases are represented by arterial stroke. Cerebral venous thrombosis is a rather rare complication with a prevalence of 0.3% (6) and usually occurs more frequently in women younger than 50 years (7). The incidence of ischemic stroke in pediatric patients positive for SARS-CoV-2 infection is relatively low. In fact, Beslow et al. found that <1% of pediatric patients hospitalized with SARS-CoV-2 had an ischemic stroke (8). Cerebral venous sinus thrombosis (CVST), as a neurological sequela, may be the consequence of the prothrombotic state triggered by COVID-19 (7, 9). Interestingly, most pediatric patients positive for SARS-CoV-2 infection who had ischemic stroke have at least one other established risk factor for stroke; thus, SARS-CoV-2 may be a contributing factor (8). However, CVST in patients with SARS-CoV-2 infection can occur in the absence of other risk factors. In fact, similar to varicella, SARS-CoV-2 may be a trigger for inflammation and pediatric cerebral arteriopathy that can lead to stroke, (8). We present the case of a 15-year-old boy admitted to the Pediatric Emergency Department of Policlinico Umberto I Hospital, “Sapienza” University of Rome (Rome, Italy) who presented with CVST and was subsequently transferred to Bambino Gesù Children's Hospital (Rome, Italy).

## Patient Information

A 15-year-old boy, obese (body weight 80 kg, height 155 cm, and BMI 33 kg/m^2^), and with mild neuromotor impairment charachterized by slow speech and clumsiness but with no familiar history of thrombosis was admitted to the pediatric emergency department of Policlinico Umberto I Hospital with dysarthria and paresthesia of the right arm and cheek. He also experienced headache, drowsiness, vomiting, and stiff neck during the last 15 days of admission. He had no fever or other symptoms. A molecular nasopharyngeal swab was performed at the onset of the symptoms by 2019 novel coronavirus real-time reverse-transcriptase PCR, and it detected the presence of SARS-CoV-2 RNA.

## Physical Examination

On admission, the patient was alert and well-oriented, with speech and motility compatible with his psychomotor development. Heart rate was 77 bpm, respiratory rate was 14 a/m, and O_2_ saturation was 100% in room air. Blood pressure was 120/83 mmHg. Neurological physical examination was normal except for dysarthria and paresthesia of the right arm and cheek. No alterations were found in the general physical examination.

## Timeline


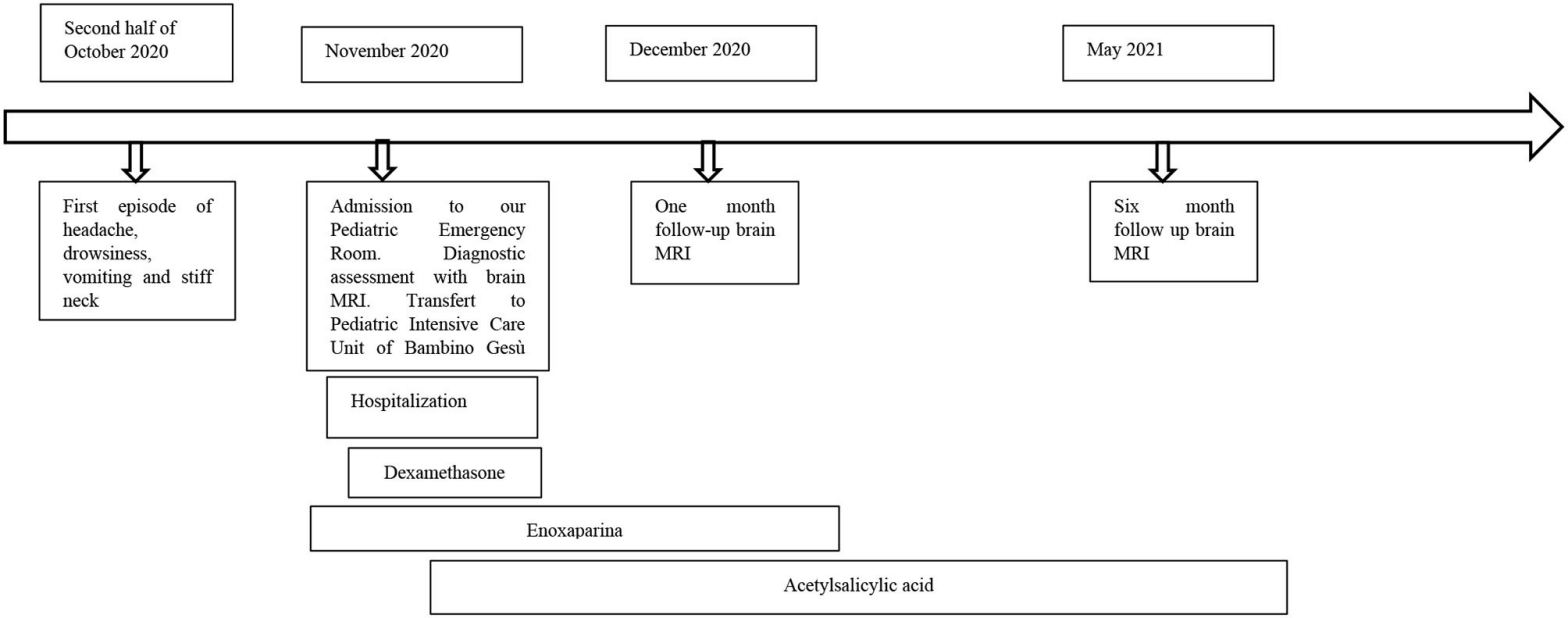


## Diagnostic Assessment

**Figure 1:**
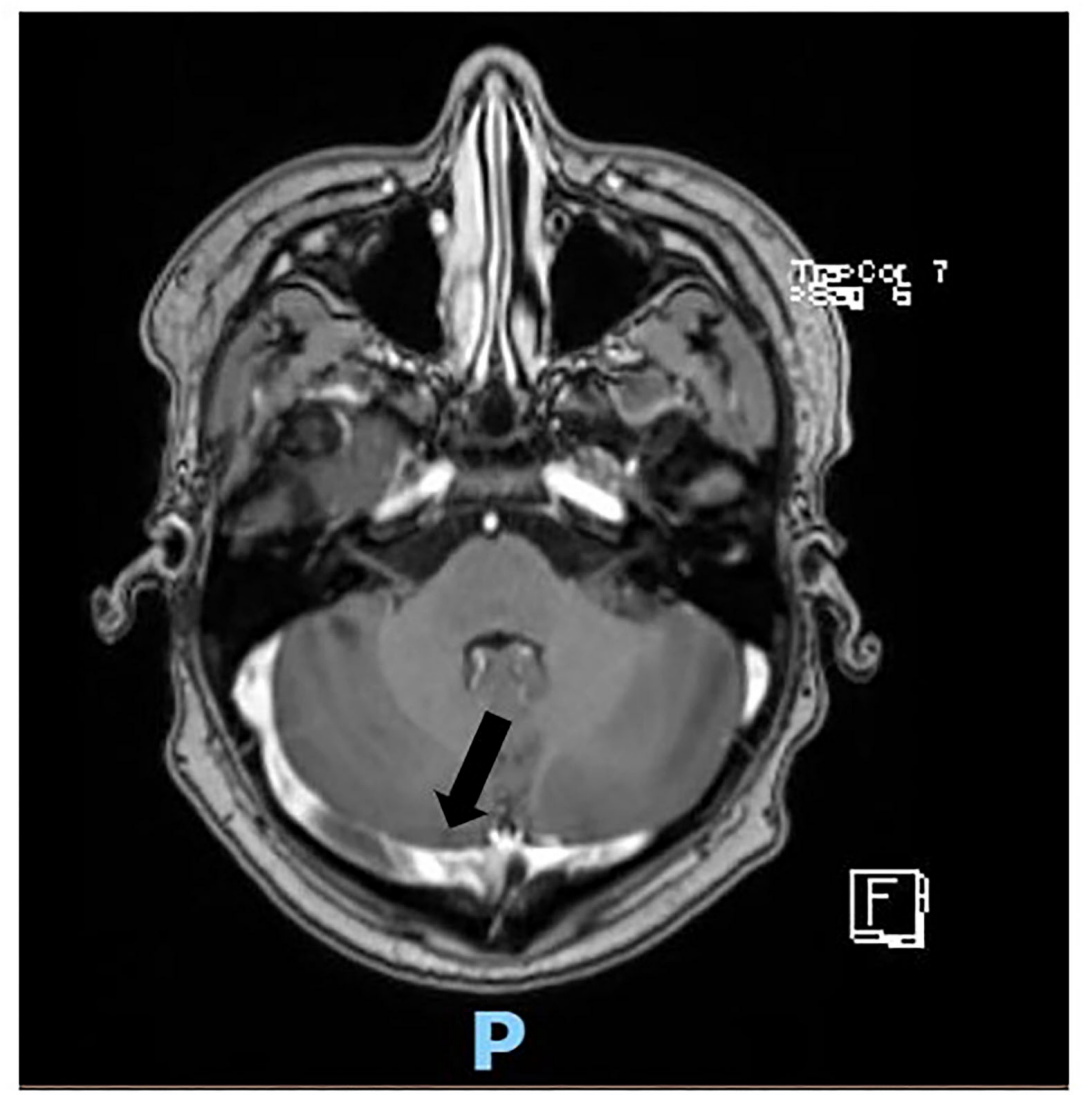
Axial scan of magnetic resonance showing thrombosis of the transverse-sigmoid sinuses of both sides (black arrow).

**Figure 2:**
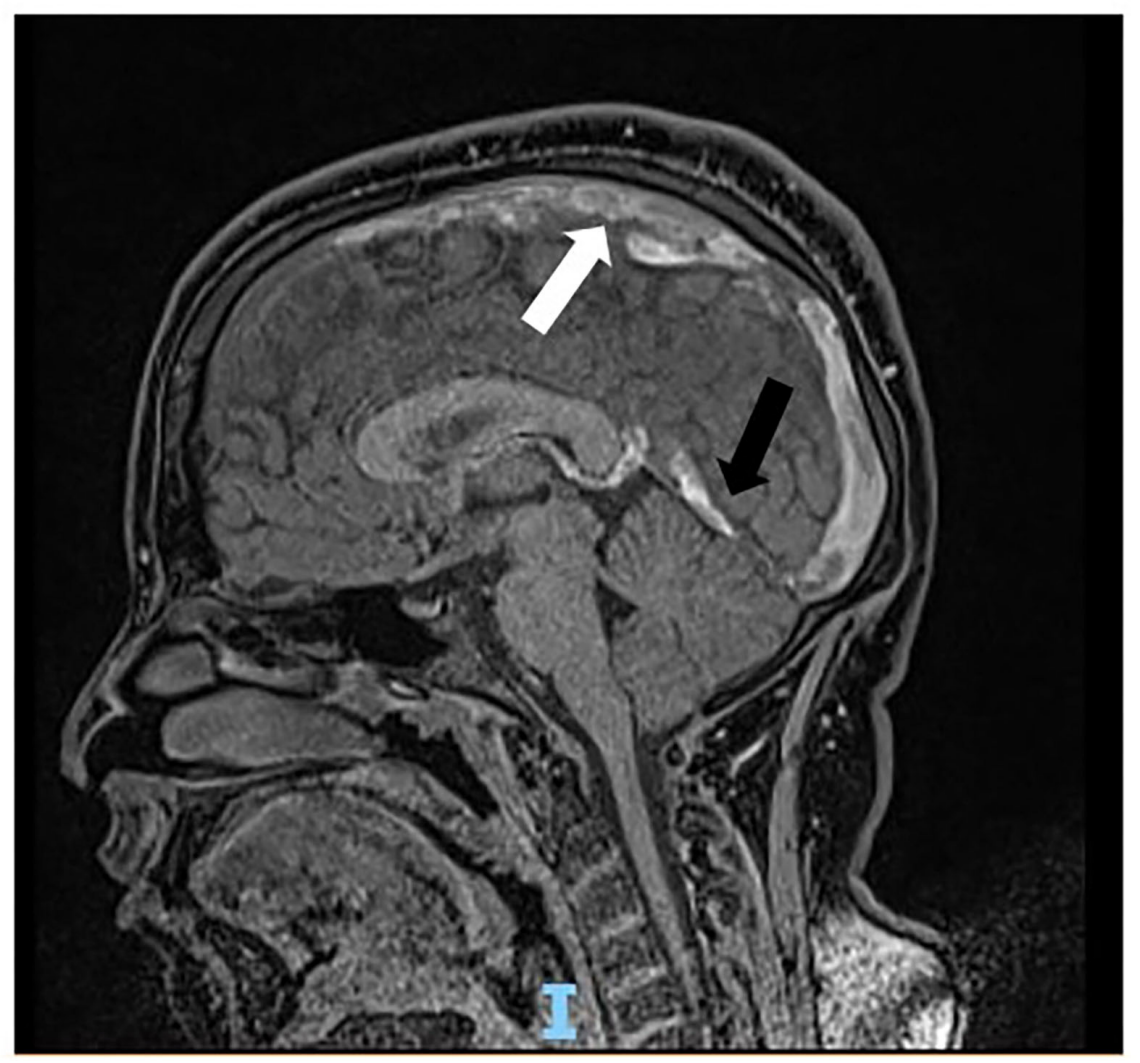
Sagittal scan of magnetic resonance showing thrombosis cast of the superior sagittal sinus (white arrow) and transverse-sigmoid sinuses (black arrow).

Soon after admission to our pediatric emergency department, in order to exclude ischemic arterial stroke or brain neoformation, a brain MRI was performed and revealed “extensive thrombotic casting in correspondence of the superior sagittal sinus, transverse-sigmoid sinuses of both sides (Figures 1, 2) and jugular veins; thrombotic manifestations at the level of cortical veins afferent to the superior sagittal sinus. Widespread congestion of medium-small caliber cortical veins. Multiple recent ischemic areoles, in subacute phase, located in both semi-oval centers at the level of right radiated rown, in subortical position at the level of the upper front and in homolateral front panel and in correspondence of the right median-paramedian splenium of the corpus callosum.”

Admission laboratory tests showed neutrophilic leukocytosis with mild increase in CRP (9,400 μg/L, n.v. 100–6,000) and fibrinogen level (492 mg/dl, n.v. 200–400), and significant elevation of D-dimer (1,697 μg/L, n.v. 0–550). He was started on therapeutic anticoagulation and admitted to the pediatric intensive care unit of Bambino Gesù Children's Hospital.

During hospitalization, a CT brain was performed that confirmed the presence of massive cerebral thrombosis and revealed mild cerebral edema. Fundoscopic exam showed papilledema, so corticosterid therapy was started. A thorough hypercoaguability workup including homocysteine, protein C, protein S, prothrombin gene mutation, Factor V Leiden genetic mutation, MTHFR gene and autoimmunity was performed and resulted in the range of normality. One week after diagnosis and the start of anticoagulant therapy, brain MRI showed no new thrombotic events and no changes in the already known ones. The neurological status of our patient improved throughout his hospital course with no more evidence of dysarthria and paresthesia. He was discharged home 25 days after admission.

## Therapeutic Intervention

He was started on subcutaneous enoxaparin 6,000 IU twice a day immediately after the diagnosis on November 12, 2020. Two days later, because of papilledema, corticosteroid therapy with dexamethasone 4 mg four times a day was started and stopped after about fifteen days. On November 23, he was started on acetylsalicylic acid 75 mg/day after hematological consultation. On the day of discharge, he was on both anticoagulant and anti-platelet therapy.

## Follow-Up and Outcomes

A brain MRI performed 1 month after discharge revealed partial resolution of the CVST, stability of ischemic areas in both semi-oval centers, and no new thrombotic lesions, so enoxaparin was stopped in December 2020. The patient continued the antiplathlet therapy until June 2021 when a brain MRI was perfomed and showed complete resolution of the CVST.

## Discussion

In the context of multisystemic involvement of COVID-19, SARS-CoV-2 could cause a wide spectrum of neurological diseases (10), but the basis of its neurotropism, neuroinvasiveness, and neurovirulence is not entirely understood. At least four potential pathogenetic mechanisms are triggered by SARS-CoV-2 infection: (1) direct neurotropic effect (e.g., anosmia), (2) systemic inflammatory response (e.g., encephalopathy), (3) immune-mediated para-infectious or postinfectious effect (e.g., GBS), and (4) vascular and prothrombotic effects (e.g., strokes) (11). COVID-19 shows a state of hypercoagulability involving disruption in the renin-angiotensin system and consequent endothelial injury characterized by reduction in Ang-1-7, binding with ACE2 receptors (12), alteration in the coagulation cascade, and coagulopathy resulting from cytokine storm activation (IL-1, IL-6, and TNF-a) (7). In particular, thrombotic complications involve the brain vasculature in 2% of cases of confirmed COVID-19 infection; most of them are represented by ischemic strokes, but there are few affected by CVST (10). CVST accounts for only 0.5–1% of all kinds of stroke and affects patients with a lower mean age (37 years) than ischemic strokes, with a female to male ratio of 3:1 (13). The main risk factors associated with CVTS are use of contraceptives, pregnancy and puerperium, genetic procoagulability conditions, malignancy, systemic inflammatory diseases and head and neck infections or injuries; obesity is not considered a main risk factor for CVST. Signs and symptoms correlated with CVTS are different according to age, sex, and time of onset and include thunderstorm or worsening headache with or without vomiting, papilledema, alterated mental status, visual complaints, encephalopathy, focal neurological deficit, and seizures. The mainstay of CVST treatment in children, similar to adults, is represented by anticoagulation with LMWH (low molecular weight heparin), UFH (unfractionated heparine) and/or warfarin even in the presence of intracranial hemorrage, according to the most recent international guidelines (14). It is reasonable to continue therapy for 3 to 6 months according to clinical condition and neuroimaging. Supportive measures for children with CVST should include appropriate hydration, control of epileptic seizures, and treatment of elevated intracranial pressure. As for the role of aspirin in management of CVST, there are still no controlled trials or observational studies discouraging its use in this kind of patients. According to the few systematic reviews available, SARS-CoV-2 infection represents a risk factor for the development of CVST. The mean age of this disease is 53.5 years which is higher than patients with CVST from other causes; its gender distribution seems balanced (50% female). (15). In addition, CVST related to SARS-CoV-2 infection appears to have an incidence approximately 3 times higher than previously published population incidence (4.5 per 100,000 vs. 1.6 per 100,000) (16) and seems to have poorer prognosis and higher mortality rate than CVTS from other risk factors (45.5 vs. 15%). A possible explanation could be the more frequent involvement of deep venous sinuses than superficial ones (16). Our clinical case represents one of the few cases described in the literature of pediatric patients positive for COVID-19 infection and have CVST (17). No respiratory or gastroentestinal involvement was described, and headache was the only clinical manifestation. The absence of other preexisting procoagulant conditions suggests that SARS-CoV-2 infection itself may represent an indipendent risk factor for CVST (18).

## Conclusion

During the COVID-19 pandemic, in patients presenting with neurological manifestations of CVST (headache, alteration of consciousness, focal deficit, or signs of endocranial hypertension), it is advisable to look for current or recent infection of SARS-CoV-2 regardless of the presence of respiratory symptoms. COVID-19-related CVST should also be suspected in pediatric patients, because, although rare in this group of patients, it can be a cause of stroke, to be recognized and treated promptly to avoid dramatic consequences both in the short and long term. Actually, the current report is one of the few pediatric CVST cases described in the literature. In our patient, ongoing SARS-CoV-2 infection represents the only prothrombotic risk factor underlying the neurological disease, once all other major causes of congenital or acquired thrombophilia are excluded. However, further studies on a wider pediatric population are needed to clarify the incidence, pathophysiology, and prognosis of patients with COVID-19-related CVST.

## Data Availability Statement

The raw data supporting the conclusions of this article will be made available by the authors, without undue reservation.

## Author Contributions

PS, AC, AF, and LM conceptualized and designed the case report, drafted the initial manuscript, and reviewed and revised the manuscript. AS and MB supervised and coordinated the manuscript and reviewed and revised it. FM, FA, and AV contributed to the conception and design of the manuscript, coordinated and supervised data collection, and critically reviewed the manuscript for important intellectual content. All authors contributed to the article and approved the submitted version.

## Conflict of Interest

The authors declare that the research was conducted in the absence of any commercial or financial relationships that could be construed as a potential conflict of interest.

## Publisher's Note

All claims expressed in this article are solely those of the authors and do not necessarily represent those of their affiliated organizations, or those of the publisher, the editors and the reviewers. Any product that may be evaluated in this article, or claim that may be made by its manufacturer, is not guaranteed or endorsed by the publisher.
